# Implementation of the Uttarakhand Tobacco Free Initiative in Schools, India, 2016

**DOI:** 10.5888/pcd18.200650

**Published:** 2021-07-29

**Authors:** Isabel Garcia de Quevedo, Rene A. Arrazola, Rajesh Yadav, Biesse D. Soura, Indu B Ahluwalia

**Affiliations:** 1 CDC Foundation, Atlanta, Georgia; 2 Centers for Disease Control and Prevention, Atlanta, Georgia; 3 Centers for Disease Control and Prevention, New Delhi, India

## Abstract

**Purpose and Objectives:**

A process evaluation, the Uttarakhand Tobacco Free Initiative (UTFI), was conducted in 49 public high schools and colleges in the state of Uttarakhand, India, to measure program implementation, provide feedback to school administrators, and identify barriers to tobacco control.

**Intervention Approach:**

UTFI aims to 1) raise awareness and provide education and tools for teachers and school administrators about the dangers of tobacco use and secondhand smoke, 2) encourage participation in student-led activities to promote tobacco-free initiatives, and 3) enforce tobacco-free school policies in the state of Uttarakhand.

**Evaluation Methods:**

We used the CDC evaluation framework to document key components and lessons learned from the UTFI. We distributed questionnaires to 71 teachers and principals in December 2016, to assess awareness of school activities and policies related to the initiative. Descriptive statistics were computed for quantitative data, and a thematic content analysis was used for qualitative data.

**Results:**

Of the 71 participants, 66 (92.9%) were aware of tobacco use policies in schools, and 63 (88.7%) agreed policies were enforced. Sixty-six participants (93.0%) said that they taught tobacco prevention-related topics, and 41 of 70 respondents (58.6%) reported that a student-led group helped to enforce tobacco-free policies in their schools. Of 69 respondents, almost all (n = 66) reported satisfaction with UTFI implementation. Challenges identified were related to tobacco products being readily accessible near school premises, lack of tobacco prevention materials, and tobacco use by school staff.

**Implications for Public Health:**

Successes of UTFI were documented by measuring different components of the process, including implementation of program activities and teacher and principal satisfaction. Results might help enhance key processes for the initiative and highlight some barriers to implementation, such as enforcing tobacco control policy off school premises. Continued efforts are needed to prevent tobacco use among young people.

**Summary**

**What is already known on this topic?**

Tobacco use in India is responsible for 1 million deaths each year. Implementation of school-based interventions may reduce student and teacher tobacco use, as part of a comprehensive approach to tobacco prevention and control.

**What is added by this report?**

Our evaluation provides lessons learned from implementation of the Uttarakhand Tobacco Free Initiative (UTFI), a school-based intervention in the state of Uttarakhand, India, to raise awareness, educate, and enforce school policies among public schools in the state.

**What are the implications for public health practice?**

Our process evaluation provides information for school administrators to improve UTFI delivery and might serve as a model for other tobacco control partners who want to implement similar school-based interventions.

## Introduction

Tobacco use, the leading cause of preventable death, results in more than 8 million deaths worldwide each year, including 1.2 million nonsmokers who are exposed to secondhand smoke (1). In India alone, tobacco use is responsible for about 1 million deaths each year (2,3). India’s most recent data on tobacco use shows that prevalence among Indian students was 14.6% in 2009 (4). In Uttarakhand, the prevalence of current tobacco use among students in grades 8 through12 was 12.2% in 2013 (20.8% boys and 0.3% girls) and 7.4% in 2016 (12.9% boys and 0.8% girls) (5,6).

As part of a comprehensive approach to tobacco prevention and control, schools are settings where programs can be implemented to prevent young people from starting tobacco use (7,8). The US Surgeon General’s Report, *Preventing Tobacco Use Among Youth and Young Adults*, concluded that multicomponent interventions that combine school-based interventions with mass media campaigns, tobacco price increases, and community-wide changes in smoke-free policies are effective in reducing the initiation, prevalence, and intensity of smoking among young people (9).

Some school-based tobacco prevention and control interventions have been successful in low-income, transitional, and high-income countries (8,10–14). These interventions have shown to be effective for young people when they include social influence models, peer support, and a duration of more than 1 year (9). The greatest impact is achieved by a multipronged approach that includes a behavioral component, policy and environmental approaches, mass media campaigns, and community-wide elements (9,10). In India, some studies that evaluated the effect of school-based tobacco interventions demonstrated positive results (13,14). A multicomponent school-based intervention that included tobacco education, advocacy, peer support, and parental involvement showed that students in the intervention group were less likely than those in the control group to smoke cigarettes or bidis during the 2-year study (15). (Bidis are small tobacco-filled leaf wraps made primarily in Southeast Asia.) Other school programs in India that focused on increasing awareness of tobacco’s harmful effects and enhancing life and advocacy skills among students and school staff reduced tobacco use among students (14,16).

## Purpose and Objectives

In 2004, India ratified the World Health Organization Framework Convention on Tobacco Control. Since then, the country has been making strides in decreasing tobacco use in the general population. India’s national comprehensive tobacco control law, The Cigarettes and Other Tobacco Products Act, 2003 (COTPA)*,* covers several topics, including protecting young people by prohibiting smoking in public places, prohibiting the sale of tobacco products to minors, prohibiting all sales of tobacco products near educational institutions, and strong pictorial health warnings on tobacco products (17). Under COTPA, the Uttarakhand state government, along with the World Lung Foundation–South Asia, adopted the Uttarakhand Tobacco Free Initiative (UTFI) to protect the state’s young people from tobacco products. UTFI implementation began in 2011 throughout all government high schools and colleges, Government Inter-Colleges, and Government Girls Inter-Colleges. The objective of UTFI is to raise public awareness about the dangers of tobacco products through education, communication, and training in schools. The aim of our cross-sectional evaluation was to assess key processes and measures related to UTFI, including 1) awareness among principals and teachers of the initiative and the activities implemented, 2) access to resources, 3) satisfaction with the initiative, 4) awareness of tobacco prohibitions in schools, and 5) barriers to implementation. Data for the evaluation were collected simultaneously with the Uttarakhand Youth Tobacco Survey (UYTS), from December 10 through December 31, 2016.

## Intervention Approach

UTFI required all government schools in the state to include the following 3 components in their programs:

**Awareness and education.** Delivery of tobacco control messages in school and at home, distribution of tobacco education materials, and training of teacher−champions (selected teachers) to implement tobacco prevention and cessation activities.
**Peer-led activities**. Creation of a student-led anti-tobacco brigade (peer-led group) to disseminate tobacco control and prevention messages and to monitor that school tobacco-use prohibition is enforced.
**School policy**. Prohibition of tobacco use on school premises for all teachers, employees, and students and ensuring closure of outlets selling tobacco products within 100 yards of educational institutions.

UTFI reached 332,634 students in 1,266 public high schools and colleges in all 13 districts of Uttarakhand. We conducted a process evaluation of UTFI by collecting cross-sectional feedback from teachers and school principals.

## Evaluation Methods

Our evaluation included principals and teacher−champions from 50 schools that participated in UYTS 2016 (5) who were also implementing UTFI. One school, which was not a part of the Uttarakhand public school system, was excluded, leaving 49 schools for analysis. Non–tobacco-consuming teachers, nominated by the schools, were assigned as teacher–champions. These teachers worked with other teachers and students to implement tobacco prevention activities in schools. Principals were responsible for the supervision of all tobacco prevention activities. Our evaluation was conducted as part of the UYTS survey and was approved by the ethical review board of the World Lung Foundation–South Asia.

### Instrument and procedures

We used the Centers for Disease Control and Prevention’s (CDC’s) Framework for Evaluation in Public Health for our evaluation, a 6-step process using both quantitative and qualitative data collection methods (18). We used a participatory approach, eliciting input from all partners throughout all phases of the evaluation process. Step 1 of the process defined the groundwork. Staff from CDC in Atlanta, the Epidemic Intelligence Service in India, and the World Lung Foundation–South Asia formed an advisory group. The advisory group worked to define the purpose of evaluation, to draft survey questions and the scope of questions, and to specify the intended use of the results. In steps 2 and 3, the advisory group also developed an evaluation plan and a logic model and developed and field-tested the data collection tool. We pilot tested UYTS on December 9, 2016, in a school in Dehradun, India’s most populated city. After pilot testing, we edited the survey and trained interviewers before initiating the full evaluation. In step 4, we implemented the evaluation plan, and the advisory group collected and sent all data to CDC for data entry, analysis, and review of findings. In steps 5 and 6, the advisory group developed a report of findings and recommendations to share with all collaborating partners in Uttarakhand.

The survey instrument, a self-administered questionnaire, consisted of 20 multiple-choice and open-ended questions. Data were collected by trained teams from South Asia’s World Lung Foundation in India and by a CDC India officer.

The survey consisted of 3 sections. The first included 7 questions on participant demographic characteristics. The second asked 2 questions about tobacco-related school policies. The third section had 11 questions related to UTFI implementation. Additionally, the survey collected information on participant awareness and perceptions about the intervention and challenges and barriers to tobacco use that the school faced.

Overall satisfaction with UTFI was measured with the question, “How would you describe your satisfaction with the implementation of the tobacco free initiative in your school?” Answers were collected by using a 5-point scale: 1 (very satisfied), 2 (somewhat satisfied), 3 (neutral), 4 (somewhat dissatisfied), and 5 (very dissatisfied), and we combined the first 2 responses (very satisfied and somewhat satisfied) into 1 category (satisfied). Attitudes about UTFI were measured with 9 questions, and answers were collected on a 5-point scale as follows: 1 (strongly agree), 2 (agree), 3 (neutral), 4 (disagree) and 5 (strongly disagree).

We recruited 2 participants per school, the teacher−champion and the school principal, for the evaluation for a total of 98 informants. We anticipated that the school principal would have high-level input on the implementation of UTFI and the teacher−champion would be best equipped to provide feedback on specific activities. All 49 schools were represented by at least 1 respondent. Of the 98 participants recruited, 71 (43 principals, 28 teacher−champions) completed the survey. The response rate was 87.0% for school principals and 57.0% for teacher–champions. Of the 49 schools represented, 22 were represented by both a principal and a teacher–champion; however, 22 schools were represented by only a school principal, and 5 by only a teacher–champion because principals were not available at the time of the survey. 

### Data analysis

Data from the questionnaires were entered into an Excel 365 (Microsoft Corporation) spreadsheet and validated to correct any data entry errors. Data were analyzed by using SAS 9.3 (SAS Institute Inc) to report descriptive statistics with frequencies and cross-tabulations. For the open-ended questions, we conducted a thematic content analysis (19). We then defined the main themes based on the survey after reading feedback from participants. Narratives were coded by 2 researchers from CDC and counter-checked by a third researcher. We developed a matrix including each theme and corresponding quotes from participants, and we resolved disagreements by consensus.

## Results

Of the 71 survey respondents, 66 (93.0%) were male. Respondents had an average of 7.4 years teaching or working in the same school, and slightly more than half of the respondents (50.7%) had been in the same school for 5 years or more. Ten (14.1%) respondents (7 teacher−champions and 3 principals) reported no knowledge of tobacco prevention programs or activities. Most respondents (93.0% principals, 75.0% teacher–champions) reported having programs in their schools that taught about tobacco use prevention, awareness of the dangers of tobacco use, and tobacco use cessation activities. Respondents that knew of tobacco prevention activities reported the following:

1. Tobacco prevention messaging activities involved regular activities that prevent students or teachers from using tobacco such as daily messages during morning prayers.
2. Tobacco cessation activities included talks prepared by students in the classroom that encourage their peers and teachers who smoke to quit, as well as activities that promote well-being.
3. Student speeches and tobacco prevention art competitions included drawing anti-tobacco images with prevention messages.
4. Outside the classroom, activities involved anti-tobacco community marches organized by students in a school and in their community.

**Figure 1:**
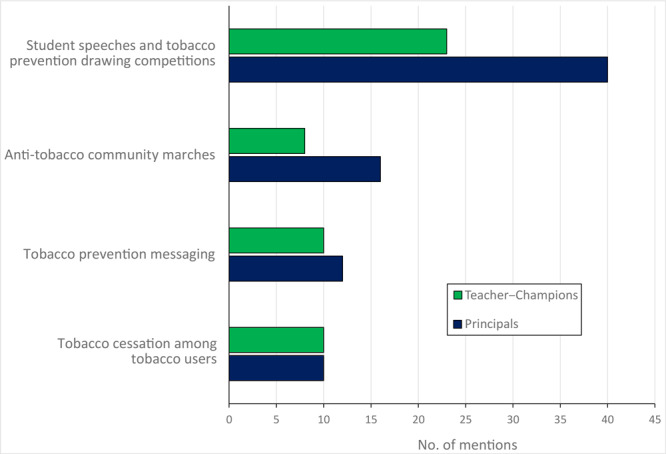
Mentions of anti-tobacco activities carried out as part of Uttarakhand Tobacco Free Initiative as reported by 71 survey respondents among school principals and teacher–champions, India, 2016. Champions were non–tobacco-consuming teachers nominated by each school.

The most reported activities by respondents in the past 12 months were student speeches and art competitions on tobacco prevention (63 mentions), followed by tobacco awareness marches (24 mentions), tobacco prevention messaging among students and in the community (22 mentions), and tobacco use cessation activities (20 mentions) (Figure 1).

**Table:** 

Type of activity	Principals	Champions
Tobacco cessation among tobacco users	10	10
Tobacco prevention messaging	12	10
Anti-tobacco community marches	16	8
Student speeches and tobacco prevention drawing competitions	40	23

When we examined data from the 22 schools in which both a principal and a teacher–champion were surveyed, most responses about activities matched between administrators and teacher–champions. Student speeches and art competitions on tobacco themes were an 81.1% match, tobacco prevention messaging among students was a 90.9% match, tobacco awareness marches were a 95.5% match, and tobacco counseling and cessation activities were an 86.4% match.

Among all teacher–champions and principals surveyed, 41 (58.6%) reported having an anti-tobacco brigade in their school; 10 (37.1%) reported no awareness of an anti-tobacco brigade*,* and 3 (4.3%) did not know if an anti-tobacco brigade existed (Table). Among schools in which both a teacher–champion and a principal responded to the survey, matched responses for this question was 90%.

**Table:** Summary of Selected Survey Results on Awareness of Tobacco Prevention Program Activities and Policies in High Schools and Colleges in the Uttarakhand Tobacco Free Initiative, India, 2016^a^

Type of Activity	Awareness of Activity	Principals, n (%) (n = 43)	Teacher–Champions,^b^ n (%) (n = 28)	All Respondents, n (%) (N = 71)
**Program implementation**				
Tobacco prevention activities took place outside classroom or school (eg, community awareness marches near students’ homes or outside school organized as a group activity)	Yes	40 (93.0)	21 (75.0)	61 (85.9)
	No	3 (7.0)	6 (21.4)	9 (12.7)
	I do not know	0	1 (3.8)	1 (1.4)
Existence of a student brigade in school^a^	Yes	24 (55.8)	17 (63.0)	41 (58.6)
	No	16 (37.2)	10 (37.0)	26 (37.1)
	I do not know	3 (7.0)	0	3 (4.3)
Frequency of teaching tobacco prevention activities during the school year	Never	4 (9.3)	1 (3.6)	5 (7.0)
	1–5 times	18 (41.9)	12 (42.9)	30 (42.3)
	6–10 times	11 (25.6)	8 (28.6)	19 (26.8)
	≥11 times	10 (23.3)	7 (25.0)	17 (23.9)
Have access to tobacco prevention teaching materials	Yes	32 (74.4)	24 (85.7)	56 (78.9)
	No	11 (25.6)	4 (14.3)	15 (21.1)
Received training on tobacco control	Yes	27 (64.3)	21 (75.0)	48 (86.6)
	No	15 (35.7)	7 (25.0)	22 (31.4)
Taught tobacco related topics	Yes	39 (90.7)	27 (96.4)	66 (93.0)
	No	4 (9.3)	1 (3.6)	5 (7.0)
**School policy on tobacco products**				
Aware that school has policy of prohibiting tobacco consumption	Yes	43 (100.0)	23 (82.1)	66 (93.0)
	No	0	5 (17.9)	5 (7.0)
	I do not know	0	0	0
There are consequences for people who consume tobacco in school^a^	Yes	39 (90.7)	24 (85.7)	63 (88.7)
	No	5 (9.3)	4 (14.3)	8 (11.3)
	I do not know	0	0	0

a The denominator is not the same for all calculations because of missing responses for some categories.

b Champions are non–tobacco-consuming teachers nominated by each school.

### Access to resources

Of the 71 respondents, 56 (78.9%; 32 [74.4%] principals, 24 [85.7%] teacher−champions) reported having access to tobacco prevention teaching materials (Table). Eighty-six percent (n = 48) of respondents reported they received training on tobacco control; 31.4% (n = 22) said they did not receive training from UTFI (15 principals, 7 teacher−champions). Ninety-three percent (n = 66) of respondents (39 principals, 27 teacher−champions) reported their school taught tobacco prevention–related topics. Forty-three percent (n = 30) reported that they taught tobacco prevention activities 1 to 5 times per year, 23.9% (n = 17) reported teaching tobacco prevention topics 11 times or more per year, and 7.0% (n = 5) (4 principals, 1 teacher–champion) reported that they never taught tobacco prevention–related topics.

### Satisfaction with the initiative

Most principals (95.4%) and most teacher–champions (96.2%) reported they were somewhat or very satisfied with the implementation of the initiative. The remaining respondents were neutral to satisfied with UTFI. No respondent reported any dissatisfaction with UTFI implementation.

**Figure 2:**
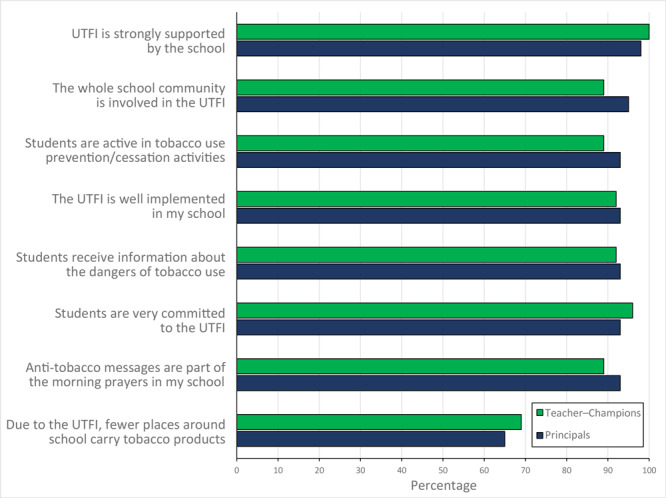
Attitudes toward Uttarakhand Tobacco Free Initiative (UTFI) among principals and teacher–champions (non–tobacco-consuming teachers), India, 2016. Responses were collected on a Likert scale with answers ranging from 1 to 5: 1, strongly agree; 2, agree; 3, neutral; 4, disagree; and 5, strongly disagree. For analysis and reporting, responses were aggregated in 2 categories: agree (strongly agree and agree) and neutral/disagree (neutral, disagree, and strongly disagree).

Most respondents agreed that UTFI was strongly supported by their school (93.0% principals, 100.0% teacher−champions), that the whole community was involved with UTFI (95.0% principals, 89.0% teacher−champions), and that students were active in tobacco use prevention and cessation activities (93.0% principals, 89.0% teacher−champions). Respondents agreed (92.8%) that UTFI was well implemented in their school. More than 30% of respondents (35.0% principals, 31.0% teacher−champions) disagreed or were neutral about the statement “Due to UTFI, fewer places around the school carry (offer) tobacco products” (Figure 2).

**Table:** 

Activity	Agree, %	
	Principals	Teacher–Champions
Due to the UTFI, fewer places around school carry tobacco products	65	69
Anti-tobacco messages are part of the morning prayers in my school	93	89
Students are very committed to the UTFI	93	96
Students receive information about the dangers of tobacco use	93	92
The UTFI is well implemented in my school	93	92
Students are active in tobacco use prevention/cessation activities	93	89
The whole school community is involved in the UTFI	95	89
UTFI is strongly supported by the school	98	100

### Awareness of tobacco prohibitions

Ninety-three percent (n = 66) of respondents reported that they were aware that their school has a policy of prohibiting tobacco use; all principals and most (82.1%) teacher–champions reported such awareness; 5 (17.9%) teacher–champions reported no awareness. Among respondents, 63 of 71 (39 principals, 24 teacher–champions) (88.7%) reported there were consequences for people who used tobacco in the schools (Table).

### Barriers to implementation

Data from the survey’s open-ended questions showed that tobacco products were still available to students through outlets located within 100 yards of the school, continuing to expose students. One teacher–champion said, “Students are exposed to various tobacco-related products off the school campus.” One principal reported, “On our school premises, we are still waiting for the anti-tobacco board.”

Another concern raised by some administrators was that teachers still used tobacco in the presence of students and noted that some teachers chewed tobacco in the form of surti or paan masala. Surti and paan masala are smokeless tobacco products prepared with lime and other spices. Using smokeless tobacco is a great obstacle to UTFI.

The open-ended responses in the questionnaire showed that overall, UTFI is viewed positively by principals and teacher–champions; however, enforcement of the policy outside school was the most frequently reported barrier by respondents reporting that tobacco products were being sold to students younger than 18 years, and that tobacco products were being sold in close proximity to schools.

## Implications for Public Health

Our evaluation showed that UTFI was received well by school administrators and that activities were well implemented. Principals and teachers seemed committed to implementing the intervention, and elements of the initiative that were not implemented correctly were outside the schools’ control, such as enforcement of tobacco product sales outside schools. Many factors might have facilitated the successful implementation of UTFI. First, the nomination of a teacher as a champion might have empowered some teachers to be more confident to lead discussions and organize activities. Previous studies showed that peers and teachers had a strong role in effective school-based interventions (11). Second, schools were encouraged to create student brigades (peer-led groups) for students to disseminate prevention messages among their peers and in the community and ensure enforcement of the school tobacco-use prohibitions. Student brigades were reported by more than half of respondents. Previous studies link the existence of a social network and peer influence to smoking behaviors among adolescents (20,21). Third, principals’ and teachers’ satisfaction levels with UTFI were high, suggesting their involvement and satisfaction made the program more likely to succeed. This finding is consistent with some studies on school interventions that show that the implementation of innovations and programs were more successful when leadership provided strong support, everyone was involved in decision making, and staff members were satisfied overall with their job and the intervention (22–24).

Most teachers and principals reported knowledge about tobacco prevention activities; however, some teacher−champions and principals that said their school had no such activities, or they were not aware of them. Additionally, responses were mismatched between some principals and teacher–champions in the same school. In some schools, teacher–champions did not report some activities that principals said were part of the school’s UTFI activities. That principals were more aware of anti-tobacco activities than teacher−champions could have been due to the high-level role principals play in school, or perhaps information might not flow well from principals to other school administrators and teachers. The finding indicates the importance of ensuring the availability of regular trainings and education for all school administrators and teachers.

India’s national tobacco control law, COPTA, prohibits the sale of tobacco products to children aged 18 years or younger and within 100 yards of educational institutions (17). Data from UYTS showed that 36.4% of students in 2013 and 56.7% of students in 2016 were aware of a policy that prohibited sale of tobacco products within 100 yards of their school. The same survey found that 16.1% of students in 2016 reported purchasing tobacco products within 100 yards of their schools, in violation of COTPA and the UTFI mandate. In our evaluation, most of teacher−champions and principals responded they were aware of the school policy. The 5 respondents who were not aware of a tobacco use prohibition were all teacher−champions. About a third of respondents disagreed that UTFI resulted in fewer places around their schools where tobacco products were sold. 

Previous studies in India show enforcement of the ban on tobacco use was the most cited barrier to implementation by school officials (25,26). With the establishment of UTFI, any observed violation must be reported to school principals or teachers, and the violator must receive a written warning and counseling. In our study, respondents were aware of a prohibition against tobacco use in schools, and lack of enforcement was observed by principals and teacher−champions; 11.3% of respondents said the school policy was not enforced. A lack of full enforcement poses a challenge to the success of the initiative to discourage smoking among students and school personnel. Taken together, our findings indicate that a comprehensive strategy that includes school administrators and law enforcement is important to successfully enforce the policy.

Since 2011 when UTFI started, 2 rounds of UYTS have been implemented: one in 2013 and the second in 2016 (5,6). The 2013 UYTS showed that 56.7% of students reported school (curriculum, teacher–champions, UTFI) as the main source of information related to anti-tobacco messages. The 2016 UYTS showed that 68.9% of students reported school as the main source of anti-tobacco messages. Our evaluation found that 7.0% of respondents reported never teaching tobacco prevention activities, although this might be due to many reasons that were not explored in our survey. Approximately 21% of respondents said they do not have access to teaching materials. Through qualitative responses, some teacher−champions said they received no training at the start of the UTFI, or that they did not have enough resources to carry out the program. Lack of materials, resources, and training were common themes reported by teacher–champions and principals in some schools, and lack of training and resources can be a critical barrier to implementation. Previous studies show that when teachers are trained properly and have the right resources, they feel more confident and better prepared to implement the curriculum and that students report improved knowledge and attitudes (27,28).

Based on our findings, tobacco control programs such as UTFI may consider the following:

- Ensure availability of educational materials necessary for tobacco prevention activities
- Provide regular training to principals and teachers on tobacco prevention strategies
- Seek opportunities to work with law enforcement to enforce adherence to school policy prohibiting sale of tobacco products within 100 yards of the school
- Conduct ongoing assessments of UTFI activities
- Work with students and student brigades to include parents and community members in tobacco prevention efforts
- Enforce school smoke-free policies on school premises for staff and students

Although our evaluation showed initial positive effects on school administrators toward UTFI, our study has several limitations. All data were self-reported, and although collected by independent evaluators, some recall and social desirability bias among responses might be present. Our study was a cross-sectional evaluation, and it was not possible to establish whether UTFI affected student behavior at any point. Because of time constraints, some in-depth interviews could not be conducted; thus, we could not collect and analyze qualitative data. Although the open-ended questions in the survey provide useful qualitative information, that cannot replace the data that might emerge through an in-depth interview. Our evaluation only targeted principals and teachers involved at the decision level in the implementation of the intervention in the school. Students involved in brigades could not be surveyed. We collected information only from teacher–champions and principals who agreed to participate in UTFI and who reported not using tobacco; therefore, we were unable to report on the perspective of principals or teachers who use tobacco. Self-selection bias might have also been possible in that nonrespondents may have been less supportive of the initiative.

Schools are an important setting to teach young people about the dangers of tobacco use. Our evaluation demonstrated the success of UTFI implementation by measuring teacher and principal awareness, knowledge, and attitudes toward the initiative. To build on this success, future efforts could focus on enforcement of existing tobacco prevention laws in Uttarakhand and ensuring school administrators have the resources to effectively implement the intervention. Moreover, even when a school-based program is implemented correctly, a comprehensive tobacco control strategy that includes evidence-based interventions is critical to prevent and reduce tobacco use among students.

## References

[1] Stanaway JD , Afshin A , Gakidou E , Lim SS , Abate D , Abate KH , ; GBD 2017 Risk Factor Collaborators. Global, regional, and national comparative risk assessment of 84 behavioural, environmental and occupational, and metabolic risks or clusters of risks for 195 countries and territories, 1990–2017: a systematic analysis for the Global Burden of Disease Study 2017. Lancet 2018;392(10159):1923–94. 10.1016/S0140-6736(18)32225-6 30496105 PMC6227755

[2] Siddiqi K , Shah S , Abbas SM , Vidyasagaran A , Jawad M , Dogar O , Global burden of disease due to smokeless tobacco consumption in adults: analysis of data from 113 countries. BMC Med 2015;13(1):194. 10.1186/s12916-015-0424-2 26278072 PMC4538761

[3] Jha P , Jacob B , Gajalakshmi V , Gupta PC , Dhingra N , Kumar R , ; RGI-CGHR Investigators. A nationally representative case-control study of smoking and death in India. N Engl J Med 2008;358(11):1137–47. 10.1056/NEJMsa0707719 18272886

[4] World Health Organization. Global Youth Tobacco Survey–India. 2009. https://www.who.int/tobacco/surveillance/gyts/en. Accessed May 21, 2021.

[5] Government of Uttarakhand, World Lung Foundation South Asia, World Health Organization, Centers for Disease Control and Prevention. Uttarakhand Youth Tobacco Survey. 2016.

[6] Government of Uttarakhand, World Lung Foundation South Asia, World Health Organization, Centers for Disease Control and Prevention. Uttarakhand Youth Tobacco Survey. 2013.

[7] Perry CL , Kelder SH , Murray DM , Klepp KI . Communitywide smoking prevention: long-term outcomes of the Minnesota Heart Health Program and the Class of 1989 Study. Am J Public Health 1992;82(9):1210–6. 10.2105/AJPH.82.9.1210 1503159 PMC1694332

[8] Thomas RE , McLellan J , Perera R . School-based programmes for preventing smoking. Cochrane Database Syst Rev 2013;(4):CD001293. 23633306 10.1002/14651858.CD001293.pub3 PMC7028068

[9] US Department of Health and Human Services. Preventing tobacco use among youth and young adults: a report of the Surgeon General. Atlanta (GA): US Department of Health and Human Services, Centers for Disease Control and Prevention, National Center for Chronic Disease Prevention and Health Promotion, Office on Smoking and Health; 2012.

[10] Thomas REMJ , McLellan J , Perera R . Effectiveness of school-based smoking prevention curricula: systematic review and meta-analysis. BMJ Open 2015;5(3):e006976. 10.1136/bmjopen-2014-006976 25757946 PMC4360839

[11] Dobbins M , DeCorby K , Manske S , Goldblatt E . Effective practices for school-based tobacco use prevention. Prev Med 2008;46(4):289–97. 10.1016/j.ypmed.2007.10.003 18093639

[12] Chen X , Fang X , Li X , Stanton B , Lin D . Stay away from tobacco: a pilot trial of a school-based adolescent smoking prevention program in Beijing, China. Nicotine Tob Res 2006;8(2):227–37. 10.1080/14622200600576479 16766415

[13] Saraf DS , Gupta SK , Pandav CS , Nongkinrih B , Kapoor SK , Pradhan SK , Effectiveness of a school based intervention for prevention of non-communicable diseases in middle school children of rural North India: a randomized controlled trial. Indian J Pediatr 2015;82(4):354–62. 10.1007/s12098-014-1562-9 25209052

[14] Sorensen G , Gupta PC , Nagler E , Viswanath K . Promoting life skills and preventing tobacco use among low-income Mumbai youth: effects of Salaam Bombay Foundation intervention. PLoS One 2012;7(4):e34982. 10.1371/journal.pone.0034982 22523567 PMC3327682

[15] Perry CL , Stigler MH , Arora M , Reddy KS . Preventing tobacco use among young people in India: Project MYTRI. Am J Public Health 2009;99(5):899–906. 10.2105/AJPH.2008.145433 19299670 PMC2667859

[16] Salaam Bombay Foundation. www.salaambombay.org. Accessed April 14, 2021.

[17] The Republic of India Ministry of Law and Justice. The Cigarette and Other Tobacco Products (Prohibition of Advertisement and Regulation of Trade and Commerce Production Supply and Distribution) Act, 2003. No. 34 of 2003. New Delhi. https://www.tobaccocontrollaws.org/legislation/country/india/laws. Accessed April 14, 2020.

[18] Centers for Disease Control and Prevention. Introduction to process evaluation in tobacco use prevention and control. 2008. https://www.cdc.gov/tobacco/stateandcommunity/tobacco_control_programs/surveillance_evaluation/process_evaluation/index.htm. Accessed March 11,2021.

[19] Guest G , MacQueen KM , Namey E . Applied thematic analysis. Los Angeles (CA): Sage Publications; 2012.

[20] Choi HJ , Smith RA . Members, isolates, and liaisons: meta-analysis of adolescents’ network positions and their smoking behavior. Subst Use Misuse 2013;48(8):612–22. 10.3109/10826084.2013.800111 23750772 PMC4355943

[21] Mercken L , Steglich C , Sinclair P , Holliday J , Moore L. A longitudinal social network analysis of peer influence, peer selection, and smoking behavior among adolescents in British schools. Health Psychol 2012;31(4):450–9. 22251218 10.1037/a0026876

[22] Pentz MA , Jasuja GK , Rohrbach LA , Sussman S , Bardo MT . Translation in tobacco and drug abuse prevention research. Eval Health Prof 2006;29(2):246–71. 10.1177/0163278706287347 16645186

[23] Marks HM , Louis KS . Does teacher empowerment affect the classroom? The implications of teacher empowerment for instructional practice and student academic performance. Educ Eval Policy Anal 1997;19(3):245–75. 10.3102/01623737019003245

[24] Gingiss PL , Gottlieb NH , Brink SG . Increasing teacher receptivity toward use of tobacco prevention education programs. J Drug Educ 1994;24(2):163–76. 10.2190/2UXC-NA52-CAL0-G9RJ 7931926

[25] Turner MM , Rimal RN , Lumby E , Cohen J , Surette A , Roundy V , Compliance with tobacco control policies in India: an examination of facilitators and barriers. Int J Tuberc Lung Dis. 2016;20(3):411– 6. 27046725 10.5588/ijtld.15.0376

[26] Kaur J , Jain DC . Tobacco control policies in India: implementation and challenges. Indian J Public Health 2011;55(3):220–7. 10.4103/0019-557X.89941 22089690

[27] Connell DB , Turner RR . School health education evaluation. The impact of instructional experience and the effects of cumulative instruction. J Sch Health 1985;55(8):324–31. 10.1111/j.1746-1561.1985.tb05657.x 3851109

[28] Gold RS , Parcel GS , Walberg HJ , Luepker RV , Portnoy B , Stone EJ . Summary and conclusions of the THTM evaluation: the expert work group perspective. J Sch Health 1991;61(1):39–42. 10.1111/j.1746-1561.1991.tb07858.x

